# Aging and Innovation: The Role of Older Adults in Research Success

**DOI:** 10.1093/geroni/igaf122.138

**Published:** 2025-12-31

**Authors:** Jodi Waterhouse, Cari Levy, Kathryn Nearing

**Affiliations:** CU Anschutz Medical Campus, Aurora, Colorado, United States; University of Colorado, Aurora, Colorado, United States; University of Colorado Anschutz Medical Campus, Denver, Colorado, United States

## Abstract

At CU Anschutz Medical Campus, older adults play a vital role in research. The Older Adult Research Specialists (OARS) program is a 14-week training initiative that empowers community-dwelling older adults with essential skills in healthcare navigation, communication, and research fundamentals. The first seven weeks focus on community health worker training, while the next phase dives into peer-to-peer recruitment, informed consent, and community engagement—ensuring older adults can effectively connect their peers with critical clinical trials. Beyond training, OARS participants serve on research teams, hold paid positions, and lead CU Anschutz Research Roadshows—immersive, educational events that bring research opportunities to urban, rural, and underserved communities across Colorado. These roadshows allow community members to learn about and engage in research without leaving their hometowns. Another innovative initiative, Living Labs in Senior Congregate Care Communities, transforms everyday spaces—dining rooms, gyms, and activity rooms—into active research hubs. Guided by the Academic Research Advisory Council (ARAC), residents help vet studies to align with their healthcare priorities. This model fosters meaningful collaboration between researchers and older adults, ensuring studies are both relevant and impactful. By embedding older adults in the research process, CU Anschutz bridges the gap between academia and community, enhancing research accessibility and inclusivity while empowering older adults as key contributors to scientific discovery.

